# Supplementary material for: Population Genomic Molecular Epidemiological Study of Macrolide-Resistant Streptococcus pyogenes in Iceland, 1995 to 2016: Identification of a Large Clonal Population with a pbp2x Mutation Conferring Reduced In Vitro β-Lactam Susceptibility
Source: J Clin Microbiol. 2020 Aug 24;58(9):e00638-20. doi: 10.1128/JCM.00638-20 (PMC7448646; doi:10.1128/JCM.00638-20)
Supplement: Supplemental file 1 [file JCM.00638-20-s0001.pdf]

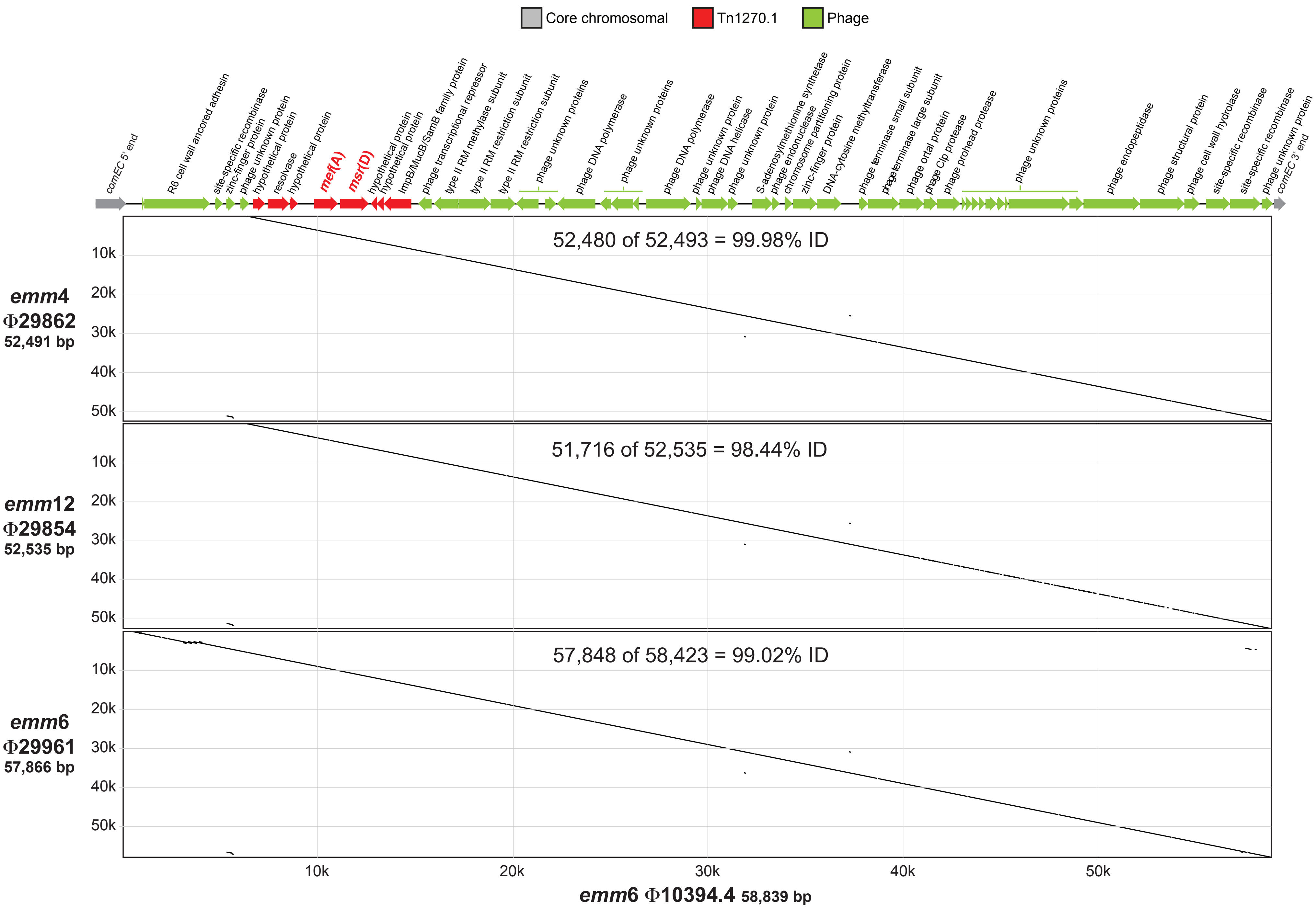

Figure S1. Composite MGEs encoding *mef*(A)-*msr*(D). Illustrated are dot matrix alignments of the *mef*(A)-*msr*(D) encoding composite MGEs identified in the Iceland erythromycin resistant *emm* type 4, 6 and 12 clonally related isolates with chimeric element 10394.4. Alignments were made with a 20 nt moving window at 95% identity. Core chromosomal, Tn1270.1 and phage gene contents of the 10394.4 gene map are colored as indicated in the index.

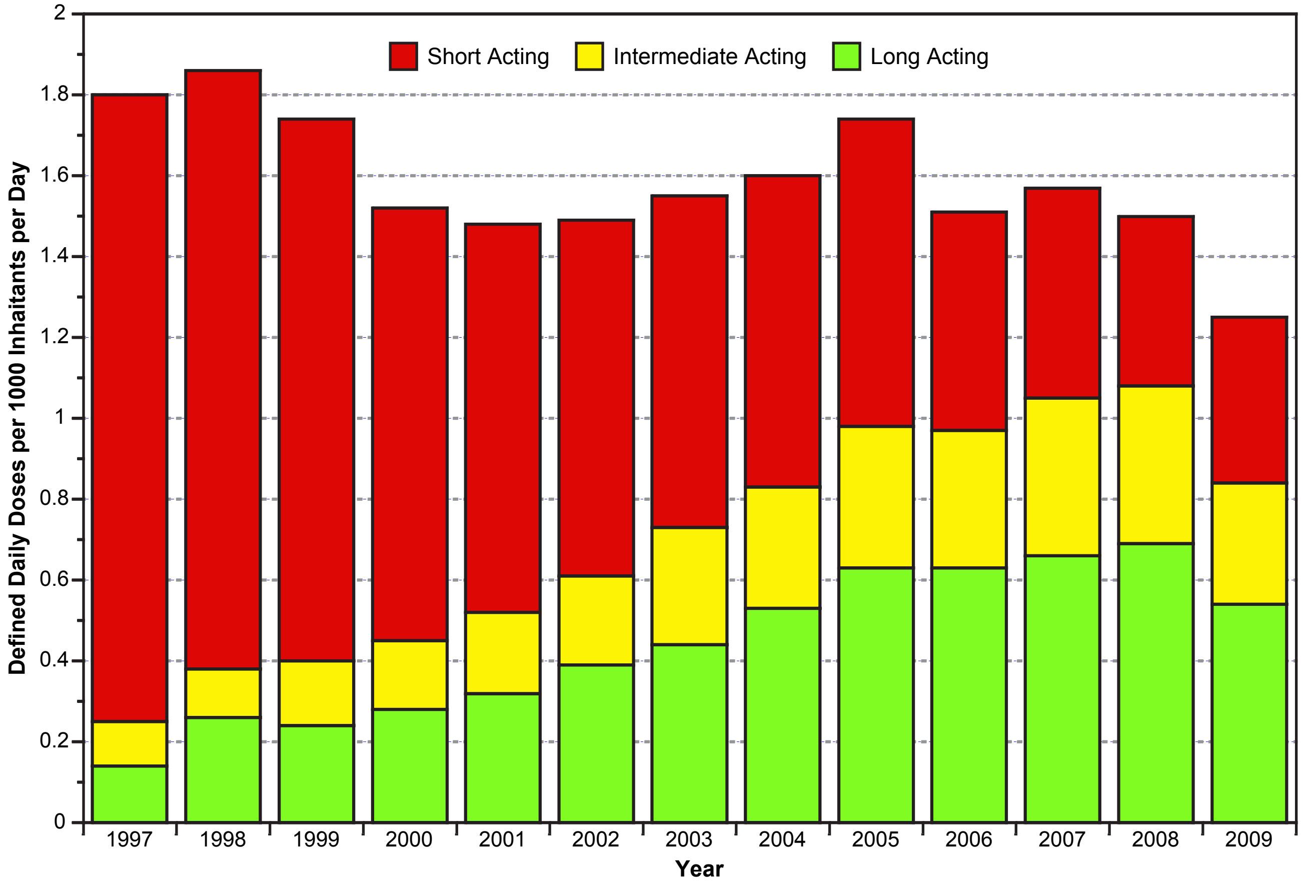

Figure S2. Macrolide antimicrobial consumption in Iceland. Illustrated is the annual outpatient macrolide consumption in mean defined daily doses per 1000 inhabitants per day. Macrolide doses are colored by mean plasma elimination half-life categoriezed as short-acting (<4 hr, such as erythromycin), intermediate-acting (>4 but <24 hr, such as clarithromycin) or long-acting (>24 hr, such as azithromycin) as indicated in the index. The data for this graph was taken from, European Surveillance of Antimcrobial Counsumption (ESAC): outpatient macrolide, lincosamide and streptogramin (MLS) use in Europe (1997-2009), Table 2 (1).

Supplemental Materials Bibliography

1. Adriaenssens N, Coenen S, Versporten A, Muller A, Minalu G, Faes C, Vankerckhoven V, Aerts M, Hens N, Molenberghs G, Goossens H, Group EP. 2011. European Surveillance of Antimicrobial Consumption (ESAC): outpatient macrolide, lincosamide and streptogramin (MLS) use in Europe (1997-2009). J Antimicrob Chemother 66 Suppl 6:vi37-45.
2. Chochua S, Metcalf BJ, Li Z, Rivers J, Mathis S, Jackson D, Gertz RE, Jr., Srinivasan V, Lynfield R, Van Beneden C, McGee L, Beall B. 2017. Population and Whole Genome Sequence Based Characterization of Invasive Group A Streptococci Recovered in the United States during 2015. mBio 8.
3. Hayes A, Lacey JA, Morris JM, Davies MR, Tong SYC. 2020. Restricted Sequence Variation in Streptococcus pyogenes Penicillin Binding Proteins. mSphere 5.
4. Kachroo P, Eraso JM, Beres SB, Olsen RJ, Zhu L, Nasser W, Bernard PE, Cantu CC, Saavedra MO, Arredondo MJ, Strope B, Do H, Kumaraswami M, Vuopio J, Grondahl-Yli-Hannuksela K, Kristinsson KG, Gottfredsson M, Pesonen M, Pensar J, Davenport ER, Clark AG, Corander J, Caugant DA, Gaini S, Magnussen MD, Kubiak SL, Nguyen HAT, Long SW, Porter AR, DeLeo FR, Musser JM. 2019. Integrated analysis of population genomics, transcriptomics and virulence provides novel insights into Streptococcus pyogenes pathogenesis. Nat Genet 51:548-559.
